# Time-Varying GPS Displacement Network Modeling by Sequential Monte Carlo

**DOI:** 10.3390/e26040342

**Published:** 2024-04-18

**Authors:** Suchanun Piriyasatit, Ercan Engin Kuruoglu, Mehmet Sinan Ozeren

**Affiliations:** 1 Tsinghua-Berkeley Shenzhen Institute, Tsinghua University, Shenzhen 518055, China; zsm22@mails.tsinghua.edu.cn; 2 Institute of Data and Information Science, Tsinghua Shenzhen International Graduate School, Shenzhen 518055, China; 3 Eurasia Earth Sciences Institute, Istanbul Technical University, 34469 Istanbul, Turkey; ozerens@itu.edu.tr

**Keywords:** sequential Monte Carlo, particle filtering, GPS time-series analysis, spatiotemporal analysis, geodetics

## Abstract

Geodetic observations through high-rate GPS time-series data allow the precise modeling of slow ground deformation at the millimeter level. However, significant attention has been devoted to utilizing these data for various earth science applications, including to determine crustal velocity fields and to detect significant displacement from earthquakes. The relationships inherent in these GPS displacement observations have not been fully explored. This study employs the sequential Monte Carlo method, specifically particle filtering (PF), to develop a time-varying analysis of the relationships among GPS displacement time-series within a network, with the aim of uncovering network dynamics. Additionally, we introduce a proposed graph representation to enhance the understanding of these relationships. Using the 1-Hz GEONET GNSS network data of the Tohoku-Oki Mw9.0 2011 as a demonstration, the results demonstrate successful parameter tracking that clarifies the observations’ underlying dynamics. These findings have potential applications in detecting anomalous displacements in the future.

## 1. Introduction

The Global Positioning System (GPS) provides permanent static displacement information which is useful in the verification of physically based models in the study of tectonic and volcanic systems [1] and complements seismological data in earthquake-related studies. It can aid in determining earthquake rupture geometry [2], estimating the time-varying distribution of fault slip [3], assessing earthquake magnitude for early warning systems [4], and detecting ground motion caused by earthquakes [5,6]. Additionally, GPS data are used to calculate velocity fields, contributing to the description of crustal deformation in various regions [7,8,9].

As the significance of GPS observations in earthquake-related studies and crustal deformation analyses becomes evident, the spatiotemporal dynamics of surface displacement from GPS network data become crucial for effective geological hazard assessment and mitigation. Previous studies have explored correlations between seismic activity and surface deformations [10], used machine learning frameworks to integrate the spatiotemporal dependencies of GPS displacements for landslide displacement prediction [11], and employed the spatiotemporal fields of GPS time-series for earthquake prediction [12].

Despite these efforts, there has been minimal attention paid to modeling the relationships among GPS measurements in a network and tracking network dynamics. This study builds upon the potential of high-rate GPS time-series data, employing the sequential Monte Carlo method, specifically particle filtering (PF), to develop a time-varying analysis of the relationships among GPS displacement time-series within a network. The aim is to uncover the network dynamics and enhance the understanding of these relationships through a proposed graph representation. Our focus is on utilizing the 1-Hz GEONET GNSS network data of the Tohoku-Oki Mw9.0 2011 earthquake as a demonstration, with results highlighting the potential of our approach for anomalous displacement detection and geological hazard assessment in the future.

## 2. GPS Data

The post-processed GPS records used in this study obtained from [13], originally composed of 1-Hz GPS displacement data from 847 GEONET [14] stations in Japan in the north–south, east–west, and up–down components. The dataset covers a period prior, during, and after the Tohoku-Oki Mw9.0 earthquake in Japan on 11 March 2011, and it was originally used in a study to detect significant ground motion from a GPS data network [6], from which the data were also shown to reliably express the ground motion caused by the propagation of seismic motion. For an illustrative purpose, a subset of these data was selected for the experiment, as discussed in Section 5.1. Additionally, the location coordinates of the GPS stations were separately obtained from [15].

## 3. Model

In this study, a network consists of a subset of *N* GPS stations. The displacement observed at any station at time epoch *t* is assumed to be related to the displacement observed at the previous time epoch t−1 of all stations including itself. A simple linear relationship for an observation at the *i*th GPS station is assumed as follows:

(1) $$\begin{array}{cc} x_{i,t} & =a_{i1,t}x_{1,t-1}+a_{i2,t}x_{2,t-1}+\cdots +a_{iN,t}x_{N,t-1}+\eta_{i,t}\\ \; & =a_{i,t}\cdot x_{t-1}+\eta_{i,t}, \end{array}$$

where $x_{i,t}$ denotes an observation of the *i*th GPS station at time epoch *t*. The vector $x_{t-1}$ denotes the observations of all GPS stations in a network at time epoch t−1. $a_{ij,t}$ are coefficients of the linear equation, which we want to recover. These coefficients reflect the influence of the previous observation at station *j* on the current observation at station *i*. Moreover, they are time-varying and can be of different values at a different time epoch *t*. Lastly, $\eta_{i,t}$ are noise terms.

Observation Equation (1) can be written in a vector form as

(2) $$\begin{array}{cc} x_{t} & =A_{t}x_{t-1}+\eta_{t}\\ \left[\begin{array}{c} x_{1,t}\\ x_{2,t}\\ \vdots \\ x_{N,t} \end{array}\right] & =\left[\begin{array}{cccc} a_{11,t} & a_{12,t} & \cdots & a_{1N,t}\\ a_{21,t} & a_{22,t} & \cdots & a_{2N,t}\\ \vdots & \vdots & \ddots & \vdots \\ a_{N1,t} & a_{N2,t} & \cdots & a_{NN,t} \end{array}\right]\left[\begin{array}{c} x_{1,t-1}\\ x_{2,t-1}\\ \vdots \\ x_{N,t-1} \end{array}\right]+\left[\begin{array}{c} \eta_{1,t}\\ \eta_{2,t}\\ \vdots \\ \eta_{N,t} \end{array}\right], \end{array}$$

where it can be noted that each *i*th row of $A_{t}$, the denoted $a_{i,t}=\left(\begin{array}{cccc} a_{i1,t} & a_{i2,t} & \cdots & a_{iN,t} \end{array}\right)$ is a hidden state vector for an observation of the *i*th GPS station at time epoch *t*, or $x_{i,t}$. Later, in Section 5.4, a graph representation based on the recovered $A_{t}$ for each time epoch is introduced.

The coefficients $a_{ij,t}$ in Equation (1) are assumed to have linear transitions from time epoch t−1 to time epoch *t* as follows:

(3) $$a_{ij,t}=a_{ij,t-1}+v_{ij,t},$$

where $v_{ij,t}$ are noise terms.

For simplicity, the state noise terms $v_{ij,t}$ in Equation (3) are assumed to be i.i.d. (independently and identically distributed) Gaussians. Conversely, the GPS observation noises $\eta_{ij,t}$ in Equation (1) have been shown not to necessarily follow Gaussian distributions [16,17,18]. These observation noises might exhibit heavier tails or other non-Gaussian characteristics. In this study, we model these observation noises $\eta_{ij,t}$, using three different distributions, i.i.d. Gaussian, i.i.d. Laplace, and i.i.d. Cauchy, and we present results for each. However, it can be noted that these noise terms can be modeled using other kinds of noise such as Gaussian mixtures [19], and alpha-stable [20] distributions, depending on the specific application requirements. This flexibility in choosing the noise terms allows for more accurate and tailored representations in various application scenarios.

Equations (1) and (3) define a model [21] widely used in time-series models, which has been applied in various fields such as computational biology [22], biogeophysics [23], brain connectivity [24], and petroleum science [25].

More precisely, our model is defined by two stochastic processes in the forms

(4) $$a_{t}=f_{t}\left(a_{t-1},v_{t}\right),$$

(5) $$x_{t}=h_{t}\left(a_{t},\eta_{t}\right),$$

where a state Equation (4) represents a process [26], in which a hidden parameter vector at time *t* depends on that of the previous time instant t−1. Equation (5) is an observation equation which is related to the hidden parameter vector $a_{t}$ of the state equation. $v_{t}$ and $\eta_{t}$ are noise terms. The intuition of the model is to capture the behaviors of an observation vector $x_{t}$ in terms of an unobserved state vector $a_{t}$.

In the case of a linear Gaussian model, where $f_{t}$ and $h_{t}$ are linear functions and the noise terms $v_{t}$ and $\eta_{t}$ are normally distributed, one of the classical methods to solve this problem is the Kalman Filter [27]. However, the aim of this study is to incorporate any existing information and model hidden parameters across any underlying distribution. Furthermore, our approach can be generalized to nonlinear observation or state equations, thereby offering enhanced flexibility and applicability across a broader spectrum of scenarios.

## 4. Sequential Monte Carlo

We propose to apply the sequential Monte Carlo method or particle filtering, a Bayesian method based on an importance sampling and resampling technique. This method is used to compute the posterior distributions of the hidden parameters, while it also allows the utilization of prior information. Importantly, this method allows nonlinearities and non-Gaussian noises in the state and observation equations. This offers flexibility to the modeling of geophysical phenomena, which may not always follow a Gaussian distribution, and deviations from the normal distribution can influence actual dynamics [28,29].

More precisely, in this study, a sequential Monte Carlo method or particle filtering (PF) is used to sequentially find the following posterior of the hidden parameter vector at each time epoch *t*, according to Bayes’ rule:

(6) $$p\left(a_{i,t}|x_{1:t}\right)=\frac{p\left(x_{t}|a_{i,t}\right)p\left(a_{i,t}|x_{1:t-1}\right)}{p\left(x_{t}|x_{1:t-1}\right)},$$

where $x_{1:t}$ denoted observations at all GPS stations from time epochs 1 to *t*, while $x_{t}$ denoted the observations at time epoch *t*. Recall that $a_{i,t}=\left(\begin{array}{cccc} a_{i1,t} & a_{i2,t} & \cdots & a_{iN,t} \end{array}\right)$ is a hidden parameter vector, which we want to recover, for an observation at the *i*th GPS station at time *t* or $x_{i,t}$.

For a Gaussian observation noise assumption, an observation has the following likelihood:

(7) $$p\left(x_{i,t}|a_{i,t}\right)=\frac{1}{{\left(2\pi \sigma_{\eta }^{2}\right)}^{1/2}}\exp\left(-\frac{{\left(x_{i,t}-{\hat{x}}_{i,t}\right)}^{2}}{2\sigma_{\eta }^{2}}\right),$$

where ${\hat{x}}_{i,t}$ is derived from $a_{i,t}$ and $x_{t-1}$ through Equation (1), and $\sigma_{\eta }$ is a standard deviation of the observation noise.

For a Laplace observation noise assumption, an observation has the following likelihood:

(8) $$p\left(x_{i,t}|a_{i,t}\right)=\frac{1}{2\beta }\exp\left(-\frac{\left|x_{i,t}-{\hat{x}}_{i,t}\right|}{\beta }\right),$$

where ${\hat{x}}_{i,t}$ is derived from $a_{i,t}$ and $x_{t-1}$ through Equation (1). β>0 is a scale parameter, and $\sqrt{2}\beta$ is a standard deviation of the observation noise.

For a Cauchy observation noise assumption, an observation has the following likelihood:

(9) $$p\left(x_{i,t}|a_{i,t}\right)=\frac{1}{\pi \gamma \left[1+{\left(\frac{x_{i,t}-{\hat{x}}_{i,t}}{\gamma }\right)}^{2}\right]},$$

where ${\hat{x}}_{i,t}$ is derived from $a_{i,t}$ and $x_{t-1}$ through Equation (1), and γ>0 is a scale parameter that determines the distribution’s spread.

Equation (6) provides the optimal Bayesian solution for the hidden parameters for Equations (4) and (5). However, the denominator in Equation (6) is intractable, and the solution often cannot be determined [30]. Particle filtering solves for the solution of the model in Equations (4) and (5) via a sampling scheme. It provides a Monte Carlo approximation for the posterior in Equation (6), using a finite number *M* of weighted samples or particles:

(10) $$p\left(a_{i,t}|x_{1:t}\right)\approx \sum_{m=1}^{M}w_{i,t}^{\left(m\right)}\delta \left(a_{i,t}-a_{i,t}^{\left(m\right)}\right),$$

where $a_{i,t}^{\left(m\right)}$ are particles, $w_{i,t}^{\left(m\right)}$ are their weights, and δ denotes the delta-Dirac function, which concentrates probability density at the particles. As the number of particles, *M*, grows and tends toward infinity, the accuracy of the approximation improves and converges towards the true distribution.

More precisely, at any time epoch *t*, the algorithm has a set of filtering particles $\left\{a_{i,t-1}^{\left(m\right)},w_{i,t-1}^{\left(m\right)}\right\}m=1\ldots M$, which represent samples from the previously estimated posterior distribution $p(a_{i,t-1}|x_{1:t-1})$. To estimate the posterior $p(a_{i,t}|x_{1:t})$ in a current iteration, we choose to sample from a proposal distribution *q*, which is perhaps convenient to sample from and approximates the target posterior distribution in some sense:

(11) $$a_{i,t}^{\left(m\right)}∼q\left(a_{i,t}|a_{i,t-1}^{\left(m\right)},x_{t}\right).$$

To ensure that particles approximate samples from the target distribution, the algorithm utilizes the sequential importance sampling method [30], where weights assigned to particles are determined by a correction factor: p/q. This is to adjust more weights to particles from critical regions, effectively reducing the overall sampling variance of the estimator. Furthermore, this particular sampling method requires fewer samples compared to alternative methods such as rejection sampling. More precisely, the importance weight [26] of a particle $a_{i,t}^{\left(m\right)}$ is assigned as

(12) $$w_{i,t}^{\left(m\right)}\propto \frac{p(a_{i,t}^{\left(m\right)}|x_{1:t})}{q(a_{i,t}^{\left(m\right)}|a_{i,t-1}^{\left(m\right)},x_{t})},$$

which, to avoid recalculation when new data arrives, is equivalent to the following sequential update [26]:

(13) $$w_{i,t}^{\left(m\right)}\propto w_{i,t-1}^{\left(m\right)}\frac{p\left(x_{i,t}|a_{i,t}^{\left(m\right)}\right)p\left(a_{i,t}^{\left(m\right)}|a_{i,t-1}^{\left(m\right)}\right)}{q(a_{i,t}^{\left(m\right)}|a_{i,t-1}^{\left(m\right)},x_{t})}.$$

The proposal distribution, *q*, should be selected based on the characteristics of the problem and the target distribution. The popular choice is a *bootstrap* filter [31], which uses the state transition density as the proposal distribution, namely to let $q\left(a_{i,t}^{\left(m\right)}|a_{i,t-1}^{\left(m\right)},x_{t}\right)=p\left(a_{i,t}^{\left(m\right)}|a_{i,t-1}^{\left(m\right)}\right)$. This results in a simplified weight update, requiring only the likelihoods as follows:

(14) $$w_{i,t}^{\left(m\right)}\propto w_{i,t-1}^{\left(m\right)}\cdot p\left(x_{i,t}|a_{i,t}^{\left(m\right)}\right).$$

The particle weights in Equation (14) are then normalized so that $\sum_{m=1}^{M}w_{i,t}^{\left(m\right)}=1$ to ensure that the weighted samples represent a valid probability distribution for the estimation of the posterior in Equation (6). The normalization [30] is as follows:

(15) $$w_{i,t}^{\prime (m)}=\frac{w_{i,t}^{\left(m\right)}}{\sum_{m=1}^{M}w_{i,t}^{\left(m\right)}}.$$

The final weighted samples ${\left\{a_{i,t}^{\left(m\right)},w_{i,t}^{\prime (m)}\right\}}_{m=1...M}$ represent samples which estimate the posterior distribution in Equation (6).

It is important to note that in high-dimensional state spaces, it can be difficult to sample particles that adequately cover the state space. This limited number of particles may struggle to represent the target distribution accurately, leading to particle weights becoming concentrated on a few particles. This problem, known as *degeneracy*, can be resolved by resampling [32], which involves replicating particles with higher weights, and removing particles with lower weights. This prevents the algorithm from being dominated by a few particles. Typically, the resampling step is triggered when $N_{eff}=\frac{1}{\sum_{m=1}^{M}{\left(w_{t}^{\left(m\right)}\right)}^{2}}$ is below a user-set threshold [26].

The particle filtering method employed in this study is summarized in Algorithm 1. It can be noted that this algorithm is applicable to real-time data. Additionally, the first set of particles are generated from a prior distribution which represents an initial belief or knowledge about the possible states of a system.
**Algorithm 1** GPS Displacement Network Learning  Input: $X=\left[x_{1},x_{2},\cdots ,x_{T}\right]\in R^{NxT}$  Output: *M* samples from $p(a_{i,t}|x_{1:t})$ for the *i*th GPS station at t=1⋯T for all i=1⋯N1:M← number of particles2:**for**i=1 to *N* **do**3:    Sample $a_{i,0}^{\left(m\right)}∼Prior\left(i\right)$ for m=1⋯M4:    Set weight $w_{i,0}^{\left(m\right)}\leftarrow 1/M$ for m=1⋯M5:**end for**6:**for** i=1 to *N* **do**7:    **for** t=1 to *T* **do**8:        Sample $a_{i,t}^{\left(m\right)}∼q\left(a_{i,t}|a_{i,t-1}^{\left(m\right)},x_{t}\right)$ for m=1⋯M (Prediction step)9:        ${\hat{x}}_{i,t}^{\left(m\right)}\leftarrow$ Equation (1) using $a_{i,t}^{\left(m\right)}$ and $x_{t-1}$ for m=1⋯M (Prediction step)10:        $w_{i,t}^{\left(m\right)}\leftarrow$ Equation (14) using ${\hat{x}}_{i,t}^{\left(m\right)}$ and $x_{t}$ for m=1⋯M (Update step)11:        ${\left\{a_{i,t}^{\left(m\right)},w_{i,t}^{\left(m\right)}\right\}}_{m=1\cdots M}\leftarrow$ Equation (15) with resampling if needed.12:    **end for**13:**end for**

## 5. Results and Discussion

### 5.1. Selected Network Data

**Figure 1:**
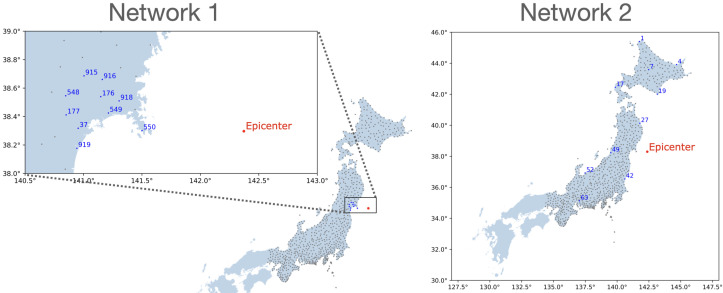
The first network (**left**) consists of 10 GPS stations, clustered near the epicenter of the earthquake. The second network (**right**) also consists of 10 GPS stations but is a sparse network. Grey dots indicate other GPS stations included in the retrieved data [13].

Two networks were selected for modeling and discussion. The first network, Network 1, is a clustered network of 10 GPS stations near the earthquake epicenter. The second network, Network 2, is a sparse network of 10 GPS stations. Locations of GPS stations in both networks are shown in Figure 1.

**Figure 2:**
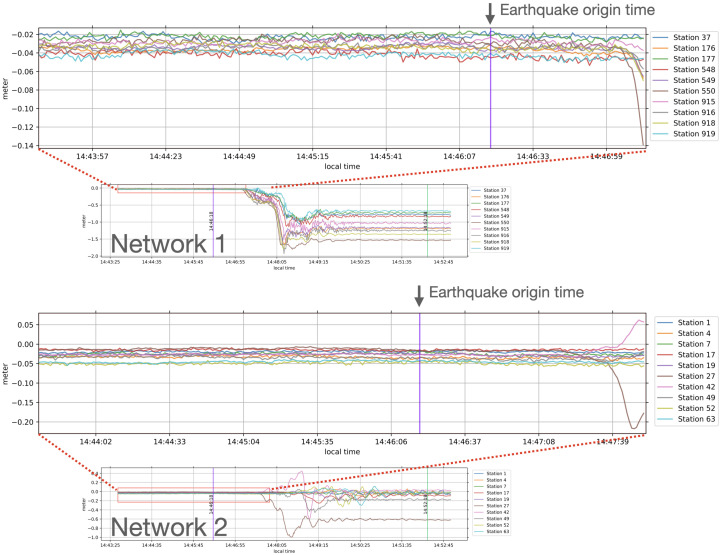
Snapshots of post-processed north displacement measurements in meters of GPS stations of the two selected networks from 14:43:38 to 14:52:58 (Japan local time) of both networks. Vertical lines in the zoomed graphs mark the earthquake’s origin time. The second vertical lines in the smaller graphs are the 6 min marks after the earthquake’s origin time.

Figure 2 shows snapshots of post-processed measurements of the north displacement in meters, retrieved from [13], from GPS stations in the two selected networks. Vertical lines mark *the earthquake’s origin time*, the time where the earthquake originates at its source, at 14:46:18 on 11 March 2011 (Japan local time) [33]. It can be noted that stations whose locations are near the earthquake epicenter experienced the shaking first; hence, significant displacements were observed at an earlier time.

Time-series were selected from GPS measurements of the north component at each station in both networks from 09:00:05 to 15:25:25 (23,131 data points) from the original 1-Hz data retrieved [13]. It can be noted that vertical displacements were not used in this study because their accuracy is usually less than that of the horizontal ones [34,35].

The number of particles used is M= 10,000. The state noise terms $v_{ij,t}$ in the state update Equation (3) are modeled as i.i.d. Gaussian distributions with zero mean, and a standard deviation of $10^{-2}$ meter (1 cm).

For observation noise, we utilize three different distributions:

First, for the Gaussian observation noise, the observation noise terms $\eta_{i,t}$ in Equation (1) are assumed to have a zero mean, and a standard deviation of $\sigma_{\eta }=10^{-2}$ m (1 cm). This value was chosen since it was reported that large coseismic ground displacement could be detected by a real-time GPS network (RTK mode) once the displacement exceeds approximately this threshold (1 cm), which represents the GPS data noise level [6].

Second, for the Laplace observation noise, the mean of the observation noise terms $\eta_{i,t}$ in Equation (1) is similarly set to zero, and the standard deviation is $10^{-2}$ m (1 cm). Consequently, this setting results in a β value of $\frac{\sigma_{\eta }}{\sqrt{2}}$ for the likelihood Equation (8).

Third, for the Cauchy observation noise, the scale parameter γ in the likelihood Equation (9) is chosen to be $10^{-2}$ m (1 cm).

The particles were initialized for the first iteration, which represents the prior information of the matrix $A_{t}$ which is a diagonal matrix with unit values along its diagonal, with zeros elsewhere, added with a normal perturbation with a zero mean and a standard deviation of $10^{-2}$ m (1 cm) to enhance the variability in the initial state estimates.

### 5.2. Modeling Results

**Figure 3:**
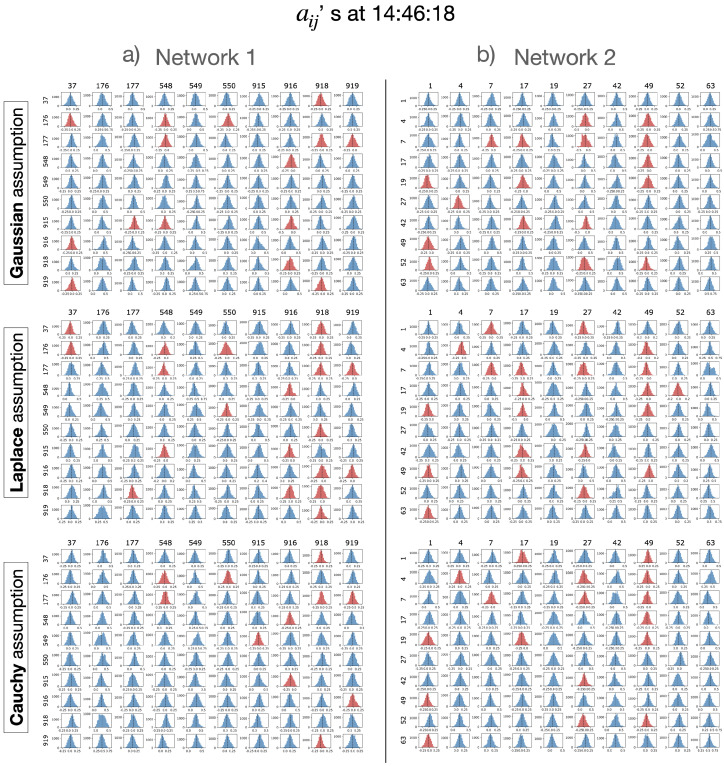
Histograms representing the distributions of particles at the earthquake’s origin time (14:46:18) of Network 1 (**a**) and Network 2 (**b**), under three observation noise assumptions: Gaussian (top row), Laplace (middle row), and Cauchy (bottom row). The arrangement of each histogram block mirrors the structure of the matrix $A_{t}$ as defined in Equation (2), with rows and columns corresponding to those of the matrix. In these histograms, the colors blue and red signify non-negative and negative particle means, respectively. A vertical line within each histogram marks the mean value of the particles, which serves as the estimate for the respective hidden parameter, $a_{i,j}$.

At each time epoch *t*, the estimated parameters are present in the form of particles. These particles serve as the basis for deriving valuable statistics, including the mean and standard deviation. Additionally, the distribution can be visually examined through representations such as histograms, which provide richer representations of the entire probability distribution. Figure 3 shows histograms of particles for the hidden parameters in the north components of Network 1 and Network 2 at one instant, under all three distributions for the observation noise. The mean value of particles for a given $a_{ij,t}$ in the matrix $A_{t}$ is considered to be the estimation for the hidden parameter $a_{ij,t}$.

The prediction residuals are calculated as the difference between the observed and estimated values. From the start of the estimation results at 9:00:05 until the earthquake’s origin time at 14:46:18, the mean values of the residuals of both networks are at zero, and the standard deviations of the residuals are 0.22 cm (Gaussian assumption) and 0.21 cm (Laplace and Cauchy assumptions) for Network 1, and 0.19 cm (Gaussian assumption) and 0.18 cm (Laplace and Cauchy assumptions) for Network 2, respectively. These figures indicate a good performance of the PF estimates, with a marginally improved accuracy observed under Laplace and Cauchy noise assumptions.

**Figure 4:**
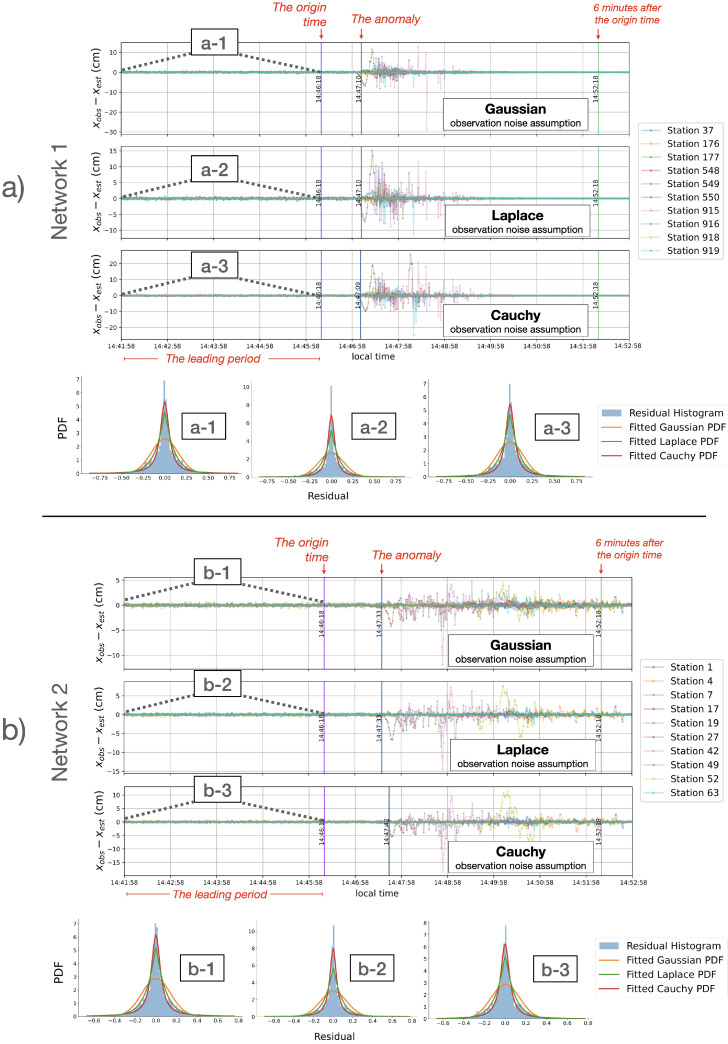
Prediction residuals in centimeters over an 11 min duration for the north components of Network 1 (**a**) and Network 2 (**b**), displayed under models with Gaussian, Laplace, and Cauchy assumptions. The first vertical line in each graph marks the earthquake’s origin time (14:46:18). The second vertical line indicates anomalies detected at 14:47:10 (for Gaussian and Laplace) and at 14:47:09 (for Cauchy) in Network 1, and at 14:47:33 (for Gaussian and Laplace) and 14:47:42 (for Cauchy) in Network 2. The third vertical line marks the 6 min point post-origin time. Above each set of residual graphs for Network 1 (**a-1**–**a-3**) and Network 2 (**b-1**–**b-3**) are histograms depicting the density of residuals during the leading 260 time points. In Network 1, histograms under all distribution assumptions show a zero mean, with standard deviations of 0.16 cm (Gaussian) and 0.15 cm (Laplace and Cauchy). In Network 2, they also present a zero mean, with standard deviations of 0.14 cm (Gaussian), 0.13 cm (Laplace), and 0.14 cm (Cauchy). Importantly, the residuals under Gaussian observation noise assumptions do not conform to a Gaussian distribution, suggesting that the observation noise deviates from Gaussian behavior.

Figure 4 shows snapshots of the prediction residuals, in centimeters, of GPS stations in Network 1 (top) and Network 2 (bottom), each under three different distribution assumptions for the observation noise. The first vertical lines in all residual graphs mark the earthquake’s origin time. Notably, following the earthquake, there is a marked fluctuation in the estimation performance, indicative of strong disturbances. This sudden fluctuation in the prediction residuals is interpreted as an anomalous event, signifying a deviation from an expected estimation. For example, in the north component of Network 1, an anomaly is detected at 14:47:10 (52 seconds after the origin time) for both Gaussian and Laplace assumptions, and at 14:47:09 for Cauchy assumption. In Network 2, an anomaly is detected at 14:47:33 (1 min and 15 seconds after the origin time) for both Gaussian and Laplace assumptions, and at 14:47:42 for Cauchy assumption. The criteria for the anomaly detection are discussed in Section 5.3.

After the anomalies, up to the 6 min mark after the origin time (as indicated by the third vertical line of each residual graph in Figure 4), Network 1 exhibited a wider range of prediction residuals (−29.1 to 12.8 cm under Gaussian assumption, −11.23 to 15.04 cm under Laplace assumption, and −24.93 to 25.81 cm under Cauchy assumption) compared to Network 2’s range (−11.9 to 4.72 cm under Gaussian assumption, −14.50 to 7.67 cm under Laplace assumption, and −18.44 to 11.58 cm under Cauchy assumption). Note that the largest observed displacements during this period were 1.93 and 0.99 m for Network 1 and Network 2, respectively. The predictions improved around the 6 min post-origin time, marked by the third vertical line in each residual graph, as expected since the disturbance caused by the earthquake started to lessen.

It can be noted that, as the number of GPS stations, *N*, in a network increases, the number of hidden parameters in the matrix $A_{t}$ will increase quadratically. Consequently, the selection of a subset of GPS stations becomes crucial and poses a challenging task.

### 5.3. Parameter Choices for Anomaly Detection

In Figure 4, anomalies are identified using specific criteria: The analysis begins by defining a *leading period* (*l*) of 260 seconds immediately preceding the earthquake’s origin time. Anomalies are those instances where the the absolute value of the prediction residual surpasses a specified *anomaly threshold* (*z*), multiplied by the standard deviation from the mean residual value calculated within the leading period. This condition must be met for a predetermined number of *consecutive counts* (*n*). The anomaly threshold *z* essentially represents the number of standard deviations by which a residual at a given time point deviates from the mean residual value of the leading period. The use of consecutive counts *n* helps to account for site-specific GPS errors. Specifically, in Figure 4, the anomalies are marked using the following parameters for both networks: *l* = 260 s window before the origin time, z=3.0, and n=5.

With these parameters, an anomaly in the east component of Network 1 is detected slightly earlier, at 14:47:05. This is consistent with expectations, as the seismic displacement from the Tohoku-Oki earthquake was first detected in the east component [6].

Our approach for identifying anomalies, which utilizes prediction residuals derived from the estimation of hidden parameters, can offer more sensitivity to the anomaly detection than if solely relying on raw displacement data. For example, when comparing anomaly detection based on prediction residuals with a straightforward approach that applies a short-term moving variance directly to the displacement observations, using the parameters previously discussed for the north component of Network 2, our method detects the anomaly the earliest at 14:47:33 (as shown in Figure 4). In contrast, with the same parameter settings, direct thresholding of the displacement observations identifies the anomaly later, at 14:47:43.

It is essential to note that the selected anomaly threshold *z*, the leading period *l*, and the consecutive counts *n* are arbitrary and can be adjusted for each network to accommodate the varying behaviors in estimation noise. For instance, in the north component of both Network 1 and 2, changing the leading period to either a 360 s or 460 s window before the earthquake’s origin time still results in detecting an anomaly within 1 s of the initial detection time, under all distribution assumptions, for the same anomaly threshold.

### 5.4. Graph Representation

The hidden parameters $a_{ij,t}$ represent relationships between GPS displacements in a network at different time epochs *t*. Instead of comparing raw values, we assess z-scores, denoted $z_{ij,t}$. A z-score is a statistical measure that indicates the number of standard deviations a data point is from the mean of its distribution. Specifically, at each time epoch *t*, a z-score of an estimated hidden parameter $a_{ij,t}$ denoted $z_{ij,t}$ is calculated as $z_{ij,t}=\frac{\left(a_{ij,t}-\mu_{t}\right)}{\sigma_{t}}$, where $\mu_{t}$ and $\sigma_{t}$ are the mean and the standard deviation of all particles $a_{ij,t}^{\left(m\right)}$, in a matrix $A_{t}$ of Equation (2).

A graphical representation of a network at time epoch *t* consists of *N* nodes, each representing a GPS station, and NxN directed edges. Directed edges from *j* to *i* represent $z_{ij,t}$. A high positive $z_{ij,t}$ reflects a positive influence of measurement of GPS station *j* on *i*, while near-zero $z_{ij,t}$ indicates proximity to the mean. Negative $z_{ij,t}$ signifies a negative influence of the measurement of GPS station *j* on *i*.

**Figure 5:**
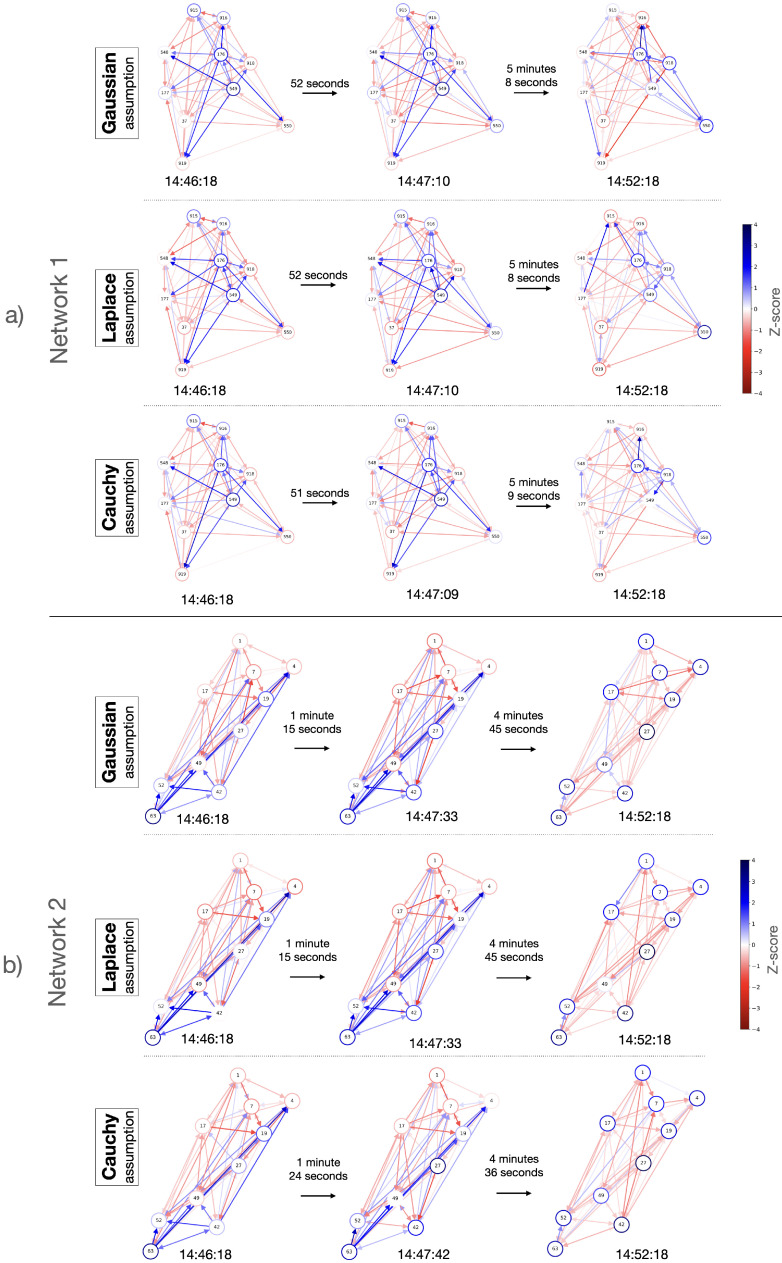
Graph representations for the north components of Networks 1 (**a**) and 2 (**b**), demonstrating hidden parameters under three observation noise assumptions: Gaussian (**top**), Laplace (**middle**), and Cauchy (**bottom**), respectively. From left to right, the columns depict the earthquake’s origin time (14:46:18), anomalies (at 14:47:10 for Gaussian and Laplace, and at 14:47:09 for Cauchy in Network 1; at 14:47:33 for Gaussian and Laplace, and at 14:47:42 for Cauchy in Network 2), and the 6 min mark from the origin time (14:52:18). The edges are color-coded, ranging from red to white to blue, corresponding to z-scores of −4, 0, and 4, respectively. For visual clarity, self-edges, connecting a node to itself, are illustrated as the borders of the nodes. The nodes are located relative to the actual positions of the corresponding GPS stations.

Figure 5 depicts graph representations of the hidden parameters for the north displacements of Network 1 and Network 2 under the three different assumptions for the observation noise, each at three distinct time instants. The graphs for the first two time instants (the origin time and the anomalies) of each network exhibit similar behaviors, noticeable by the similar colors of their edges. However, at the third time instant shown, both networks display graphs distinct from those in the previous two time instants. This divergence is understandable since, at the marked anomalies of both networks (center column), the networks began to undergo changes, reflected by high estimate residuals (noticeable in Figure 4), caused by the disturbance from the earthquake that the model did not capture well initially. Subsequently, at the third time instant (the rightmost column), the model learns and presents different behaviors, as indicated by graphs with distinctly different edge colors. It can be noted that in Network 2, at the third time instant shown, each GPS station’s measurement heavily relies solely on its own previous measurements. This may be due to the highly sparse locations of the stations and hence less dependency after the disturbance from the earthquake. Additionally, it can be noted that the Cauchy observation noise assumptions lead to sparser networks with less significant branches, as shown by the lighter colors of the graph edges.

The proposed graph representation of a network enables the tracking of hidden parameters in a compact manner, with edges defined using the z-scores of the estimated parameters. This representation should also aid the understanding of networks following weaker earthquakes, whose hidden parameters are expected to fluctuate more subtly. Other potential graph representations include a graph whose edges are defined based on the amount of change in the parameters relative to those in the previous time step.

## 6. Conclusions

We showcased the capabilities of a sequential Monte Carlo method, specifically particle filtering, in tracking changes in the relationships among GPS measurements in a network before, during, and after the disturbance caused by an earthquake. Our proposed model effectively captures the time-varying behaviors of the network, which can be useful for anomalous displacement detection. The proposed graph representations aid in understanding and facilitate the tracking of network dynamics. The versatility of the method allows for extensions to model other time-varying geodetic data networks, and it facilitates the adoption of different model equations and assumptions. In the future, regarding this work, we will also consider other distribution models [36] and nonlinear state and observation models.

## Data Availability

The original code presented in the study is openly available at https://github.com/SuchanunP/pf_gps_dynamics/ (accessed on 2 April 2024). The animated graph representations of Network 1 under three distribution assumptions for the observation noise are included in the Supplementary Materials, and further inquiries can be directed to the corresponding author.

## Supplementary Materials

The following supporting information can be downloaded at: https://www.mdpi.com/article/10.3390/e26040342/s1.
